# Small bowel perforation secondary to swallowed denture impaction at an NSAID-induced ileal diaphragm

**DOI:** 10.1093/jscr/rjag763

**Published:** 2026-09-07

**Authors:** Sukhbir Sira, Andrew Webster, Craig Mackay

**Affiliations:** General Surgery Department, Aberdeen Royal Infirmary, Foresterhill Road, Aberdeen, Scotland AB25 2ZN, United Kingdom; General Surgery Department, Aberdeen Royal Infirmary, Foresterhill Road, Aberdeen, Scotland AB25 2ZN, United Kingdom; Medical Education, University of Aberdeen, Kings College, Aberdeen, Scotland AB24 3FX, United Kingdom; General Surgery Department, Aberdeen Royal Infirmary, Foresterhill Road, Aberdeen, Scotland AB25 2ZN, United Kingdom

**Keywords:** diaphragm disease, NSAID enteropathy, meloxicam, small bowel perforation, foreign body impaction, Crohn’s disease mimic

## Abstract

Diaphragm disease of the small bowel is a rare but well-documented complication of prolonged nonsteroidal anti-inflammatory drug (NSAID) use. Characterized by circumferential mucosal diaphragms, the disease often presents as small bowel obstruction but can also lead to perforation. We report a case of small bowel perforation caused by a swallowed partial denture that became lodged at an ileal diaphragm in a patient with a history of long-term NSAID use. The diagnosis was confirmed histologically following a right hemicolectomy with primary stapled anastomosis. This case highlights the importance of considering diaphragm disease in patients on chronic NSAID therapy who present with signs of bowel obstruction or perforation, even when alternative diagnoses, such as Crohn’s disease, are initially suspected.

## Introduction

First described by Lang *et al*. in 1988, diaphragm disease is a rare but recognized complication of long-term nonsteroidal anti-inflammatory drug (NSAID) use, characterized by thin, concentric mucosal bands in the small intestine, typically in the ileum [1]. These diaphragms can lead to strictures, obstruction, and, in rare cases, perforation [1, 2]. NSAIDs induce gastrointestinal injury primarily by inhibiting cyclooxygenase-1 (COX-1), impairing mucosal defence mechanisms and reducing blood flow, contributing to ulceration and fibrotic healing [3, 4]. We present a unique case in which a swallowed partial denture became lodged at an ileal diaphragm, causing small bowel perforation in a patient with presumed Crohn’s disease.

## Case report

A man in his fifties presented with a 2-day history of progressively worsening abdominal pain. The pain initially began in the right iliac fossa and became generalized over several days, with radiation to bilateral groin, left lower abdomen, and the back. He reported associated anorexia but no preceding diarrhoea or rectal bleeding.

His past medical history included a presumed diagnosis of Crohn’s disease in 2013, traumatic vertebral fractures, and asthma. The 2013 diagnosis was based on computed tomography (CT) imaging demonstrating terminal ileal thickening and capsule endoscopy revealing ulceration with capsule retention within a strictured segment. No histological confirmation was obtained at that time. He was not commenced on long-term corticosteroids, immunomodulators, or biologic therapy and was not receiving active inflammatory bowel disease treatment at the time of presentation.

He had been taking meloxicam regularly for ~6 years for chronic musculoskeletal pain related to vertebral fractures. Prior to commencing meloxicam, he had taken diclofenac regularly for ~7 years. He was prescribed apixaban for previous deep vein thrombosis of the arm.

On admission, haemoglobin was 112 g/l, white cell count was 17 × 10^9^/l, neutrophils were 12 × 10^9^/l, and C-reactive protein was 378 mg/l. A venous blood gas revealed: pH 7.42, lactate 2.4 mmol/l, pO_2_ 6.87 kPa, pCO_2_ 4.96 kPa, and HCO_3_ 24.3 mmol/l.

**Figure 1:**
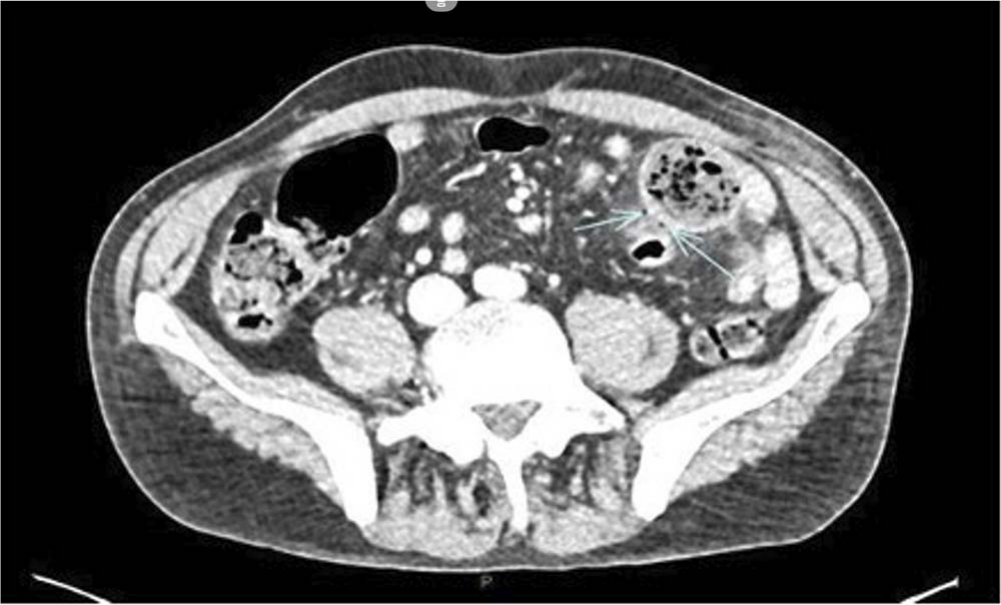
Contrast-enhanced CT abdomen demonstrating dilated small bowel loops proximal to a transition point in the distal ileum.

**Figure 2:**
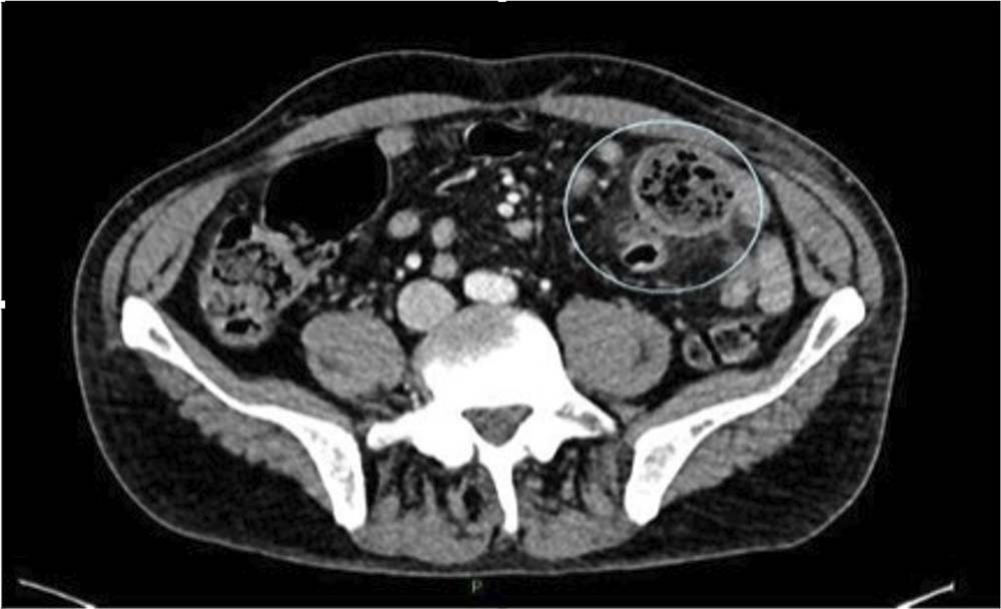
Contrast-enhanced CT abdomen demonstrating distal ileal mural thickening with localized extraluminal gas, consistent with contained perforation.

Contrast-enhanced CT of the abdomen and pelvis demonstrated distal small bowel dilatation with small bowel faecalization, with an abrupt transition point in the distal ileum, mural thickening, and localized extraluminal gas consistent with contained perforation (Figs 1 and 2).

Differential diagnoses initially included Crohn’s-related small bowel stricture, small bowel obstruction, and cryptogenic multifocal ulcerous stenosing enteritis.

Emergency laparotomy revealed a large intra-abdominal collection and a markedly abnormal terminal ileum. The affected bowel was oedematous, dusky, and non-viable, with inflammation extending to the ileocaecal junction. Given the extent of pathology and proximity to the ileocaecal junction, an ileocaecal resection with primary side-to-side stapled anastomosis was performed.

**Figure 3:**
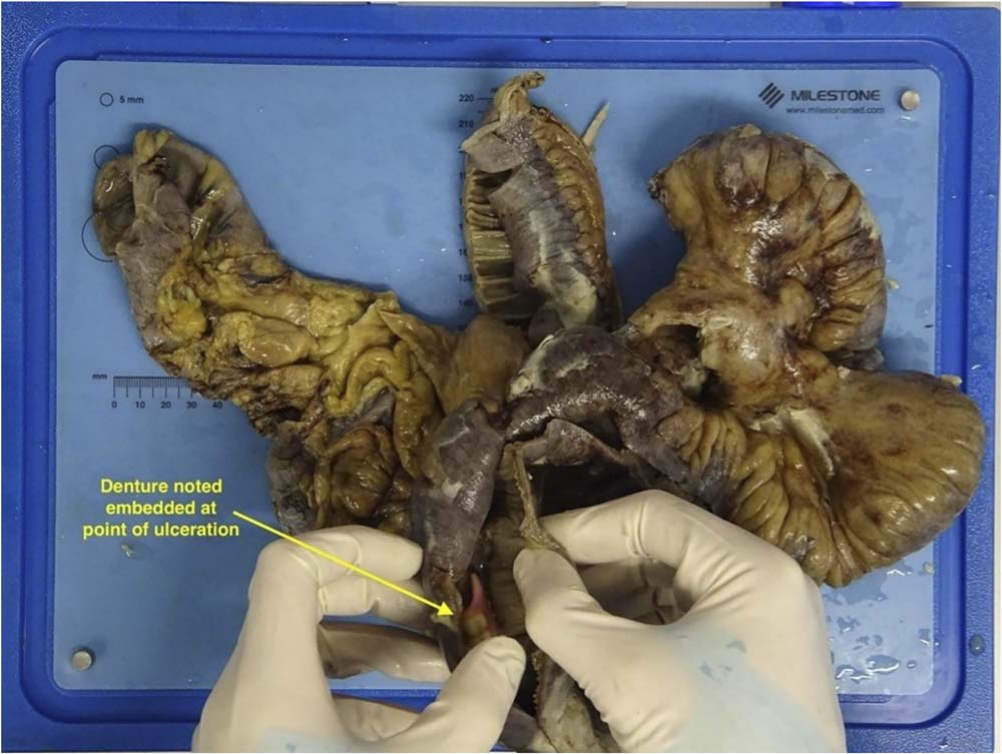
Gross specimen demonstrating the partial denture impacted within the terminal ileum adjacent to the ulcerated perforation site.

**Figure 4:**
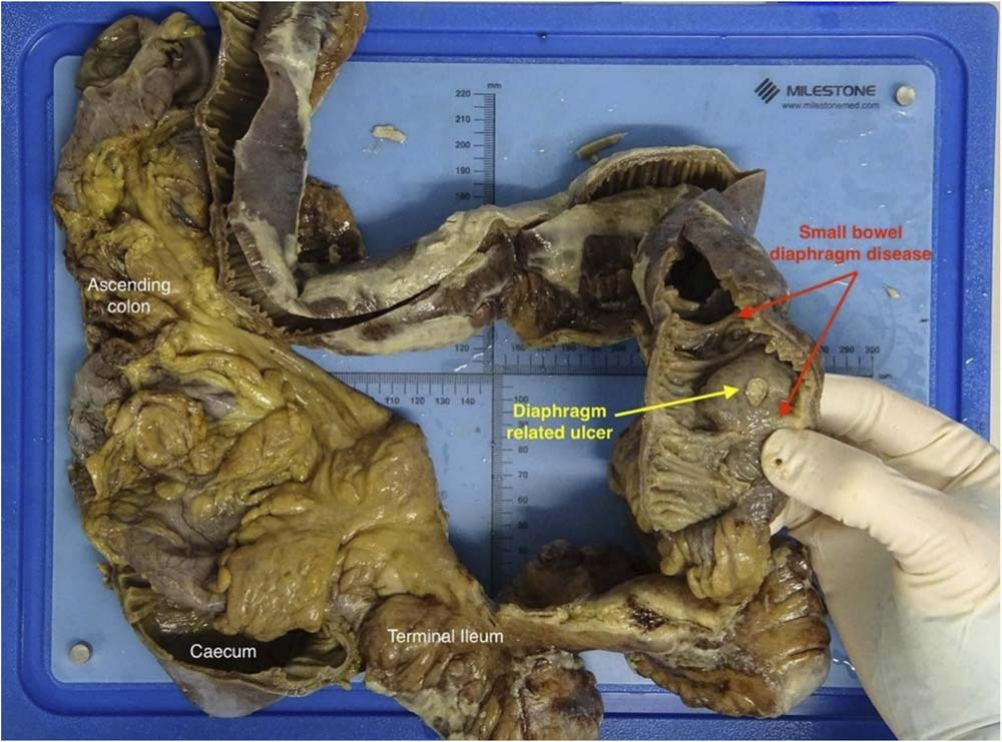
Gross specimen demonstrating multiple diaphragm-like ileal strictures with associated ulceration, characteristic of NSAID-induced diaphragm disease.

Histopathological findings were as follows. A 2.7 × 2.8 × 1.7 cm partial denture was identified within the terminal ileum, embedded adjacent to a 1 cm ulcer. This ulcer lay immediately proximal to a diaphragm-like stricture (Figs 3 and 4). Multiple diaphragm-like strictures were identified within the resected specimen. Histopathological examination confirmed diaphragm disease, demonstrating characteristic mucosal folds with submucosal fibrosis and associated ulceration. The ulcer was associated with transmural neutrophilic inflammation and serosal exudate, consistent with perforation and localized peritonitis. Background small bowel mucosa was otherwise normal, with no granulomas, crypt architectural distortion, or features suggestive of inflammatory bowel disease. No additional macroscopic diaphragms were identified beyond the resected segment at index laparotomy.

Ten days postoperatively, the patient developed worsening abdominal pain and inflammatory markers. Re-look laparotomy identified an anastomotic leak with localized faecal contamination. Segmental small bowel resection with end ileostomy formation was performed, with the distal bowel closed as a stump. Given the recent anastomotic failure, contamination, and concern regarding bowel viability, an end ileostomy was favoured over loop diversion. The patient recovered following reoperation and remains under outpatient follow-up.

## Discussion

This case illustrates a rare instance of small bowel perforation due to a swallowed denture becoming lodged in an NSAID-induced ileal diaphragm. Multiple reports document NSAID-associated small bowel injury and diaphragm-like strictures, including with relatively COX-2 selective agents such as meloxicam, suggesting that relative COX-2 selectivity does not eliminate enteropathic risk [1, 2]. Histopathological examination confirmed diaphragm disease, demonstrating characteristic diaphragm-like mucosal architecture with submucosal fibrosis, without granulomas or chronic transmural inflammation. Although limited mesenteric fat encroachment was noted macroscopically, definite fat wrapping was not identified histologically. The absence of histological confirmation in 2013, repeated normal colonic histology, lack of ongoing inflammatory bowel disease-directed therapy, and prolonged NSAID exposure support NSAID-induced diaphragm disease as the more likely underlying pathology. Capsule retention previously attributed to Crohn’s disease may retrospectively represent undiagnosed diaphragm disease. Diaphragm disease should be considered in NSAID users presenting with obstruction, perforation, or unexplained abdominal symptoms [3–7]. A 2024 report described diaphragm disease of the terminal ileum as ‘the great imitator’, highlighting overlapping CT features, namely, segmental thickening, hyperenhancement, and peri-enteric stranding and the risk of misclassification as inflammatory bowel disease without histological confirmation [8]. This case highlights how diaphragm disease may mimic Crohn’s disease radiologically; definitive diagnosis often relies on histopathological assessment. Foreign body impaction in areas of pathological narrowing, such as strictures or diaphragms, is a known mechanism of small bowel perforation, albeit uncommon [9, 10]. From a surgical perspective, where multifocal diaphragms, uncertain bowel viability, or ileocaecal involvement is encountered and the aetiology remains uncertain intraoperatively, a wider resection may be justified to secure margins and reduce anastomotic risk. This approach should be interpreted cautiously, acknowledging the absence of randomized evidence and the need to balance extent of resection against operative morbidity [11].

## Consent

Written informed consent was obtained from the patient for publication of this case report and accompanying images.
